# Prevalence and correlates of psychoactive substance use in domestic and foreign university students

**DOI:** 10.1192/j.eurpsy.2024.525

**Published:** 2024-08-27

**Authors:** E. L. Nikolaev, S. S. Fakhraei

**Affiliations:** ^1^Department of Social and Clinical Psychology; ^2^Medical Faculty, Ulianov Chuvash State University, Cheboksary, Russian Federation

## Abstract

**Introduction:**

Use of psychoactive substances is a risk factor for mental health. Studying the peculiarities of using psychoactive substances by university students is extremely important for organizing preventive health care

**Objectives:**

To specify the frequency of smoking and alcohol drinking, as well as the peculiarities of the correlational interconnections, in domestic and foreign university students

**Methods:**

The survey covered 546 undergraduate domestic and foreign university students of both genders and different religious backgrounds. As a tool, we used the Sociocultural Health Questionnaire (E. Nikolaev)

**Results:**

It has been revealed that domestic students smoke cigarettes and hookahs surely more often (p=.01) than foreign students (30.49% vs 19.08%). It is obvious that they also more often (p=.01) use electronic cigarettes or vaping drugs (25.24% vs 12.86%) and alcohol (54.42% vs 9.96%). Students in both groups denied using other psychoactive substances. Foreign students reveal positive correlational interconnections between smoking and alcohol drinking (r=.44), while there is no evidence of such interconnections in domestic students. Both groups show valid interconnections between the frequency of smoking and the level of stress (r=.15 и r=.17 correspondingly), the frequency of smoking and monthly financial expenses (r=.21 и r=.22 correspondingly). With domestic students, vaping negatively correlates with exercising in gyms (r=-.12), with foreign students it directly correlates with bodybuilding supplements consumption (r=.15). Those foreign students who drink alcohol more often point to the necessity of having a psychologist in the university (r=.13).

**Conclusions:**

The revealed general and specific factors associated with domestic and foreign students’ use of psychoactive substances call for the necessity of developing culturally differentiated preventive programs

**Disclosure of Interest:**

None Declared

